# Supplementary material for: Modelling Skylarks (Alauda arvensis) to Predict Impacts of Changes in Land Management and Policy: Development and Testing of an Agent-Based Model
Source: PLoS One. 2013 Jun 6;8(6):e65803. doi: 10.1371/journal.pone.0065803 (PMC3675089; doi:10.1371/journal.pone.0065803)
Supplement: Supporting Information S4 — The skylark ODdox as a zipped archive. (ZIP) [file pone.0065803.s004.zip › Skylark_ODdox/functions_0x74.html]

ALMaSS Skylark ODdox: Class Members


ALMaSS Skylark ODdox
 2.0


- Main Page
- Related Pages
- Classes
- Files

- Class List
- Class Index
- Class Hierarchy
- Class Members

- All
- Functions
- Variables
- Typedefs

- a
- b
- c
- d
- e
- f
- g
- h
- i
- j
- k
- l
- m
- n
- o
- p
- r
- s
- t
- u
- v
- w
- x
- y
- z
- ~

Here is a list of all class members with links to the classes they belong to:

### - t -

- TALMaSSObject()
  : TALMaSSObject
- TAnimal()
  : TAnimal
- Territories
  : SkTerritories
- Terrs
  : Skylark\_struct
- Test()
  : Pesticide
- TestCropManagement()
  : Landscape
- TestFile
  : Population\_Manager
- TestFile2
  : Population\_Manager
- TestNestPossibility()
  : skTTerritory
- TheArray
  : Population\_Manager
- TheBreedingFemalesProbe()
  : Population\_Manager
  , Skylark\_Population\_Manager
- TheBreedingSuccessProbe()
  : Population\_Manager
  , Skylark\_Population\_Manager
- TheCIPEGridOutputProbe()
  : Skylark\_Population\_Manager
  , Population\_Manager
- TheFledgelingProbe()
  : Population\_Manager
  , Skylark\_Population\_Manager
- TheGeneticProbe()
  : Population\_Manager
- TheLandscape
  : SkTerritories
- TheNWordOutputProbe()
  : Population\_Manager
  , Skylark\_Population\_Manager
- TheProbe
  : Population\_Manager
- TheReallyBigOutputProbe()
  : Population\_Manager
- TheRipleysOutputProbe()
  : Population\_Manager
  , Skylark\_Population\_Manager
- TheSkylarkTerrs
  : Skylark\_Population\_Manager
- TheSkylarkTerrsSupply\_quality()
  : Skylark\_Population\_Manager
- TheSkylarkTerrsSupply\_size()
  : Skylark\_Population\_Manager
- TheSkylarkTerrsSupply\_x()
  : Skylark\_Population\_Manager
- TheSkylarkTerrsSupply\_y()
  : Skylark\_Population\_Manager
- Tick()
  : Calendar
  , LE
  , Landscape
  , Pesticide
  , RodenticideManager
  , Weather
  , SkTerritories
- TickHour()
  : Landscape
  , Calendar
- TickMinute()
  : Calendar
  , Landscape
- ToggleCattleGrazing()
  : VegElement
  , LE
- TogglePigGrazing()
  : LE
  , VegElement
- Tov
  : PermCropData
- Trace()
  : LE
- Track()
  : Track
- TranslateCropCodes()
  : Farm
- TranslateEleTypes()
  : LE\_TypeClass
  , Landscape
- TranslateVegTypes()
  : LE\_TypeClass
  , Landscape
- Trial\_Control()
  : Farm
- Trial\_PesticideTreat()
  : Farm
- Trial\_PesticideTreat\_GS()
  : Farm
- Trial\_ToxicControl()
  : Farm
- TSkylarkList()
  : TSkylarkList< T >
- TurnTheWorld()
  : Landscape
- TwinMapAddToMain()
  : Pesticide
- TwinMapClear()
  : Pesticide
- TwinMapCopyToMask()
  : Pesticide
- TwinMapDiffusion()
  : Pesticide
- TwinMapSpray()
  : Pesticide
- TwinMapSprayCorrectBorders()
  : Pesticide
- TwinMapSprayPixel()
  : Pesticide


- Generated on Thu Jan 10 2013 13:15:39 for ALMaSS Skylark ODdox by
   1.8.1.1
